# Regenerative Medicine and Angiogenesis; Challenges and Opportunities

**DOI:** 10.34172/apb.2020.061

**Published:** 2020-08-09

**Authors:** Mozhgan Jahani, Davood Rezazadeh, Parisa Mohammadi, Amir Abdolmaleki, Amir Norooznezhad, Kamran Mansouri

**Affiliations:** ^1^Medical Biology Research Center, Health Technology Institute, Kermanshah University of Medical Sciences, Kermanshah, Iran.; ^2^Molecular Medicine Department, Faculty of Medicine, Kermanshah University of Medical Sciences, Kermanshah, Iran.; ^3^Applied Cell Sciences Department, Faculty of Medicine, Shahid Beheshti University of Medical Sciences, Tehran, Iran.; ^4^Department of Anatomical Sciences, Medical School, Kermanshah University of Medical Sciences, Kermanshah, Iran.

**Keywords:** Regenerative medicine, Angiogenesis, Stem cells, Scaffold

## Abstract

Blood vessel development is one of the most prominent steps in regenerative medicine due to the restoration of blood flow to the ischemic tissues and providing the rapid vascularization in clinical-sized tissue-engineered grafts. However, currently tissue engineering technique is restricted because of the inadequate *in vitro/in vivo* tissue vascularization. Some challenges like as transportation in large scale, distribution of the nutrients and poor oxygen diffusion limit the progression of vessels in smaller than clinically relevant dimensions as well *in vivo* integration. In this regard, the scholars attempted to promote the vascularization process relied on the stem cells (SCs), growth factors as well as exosomes and interactions of biomaterials with all of them to enable the emergence of ideal microenvironment which is needed for treatment of unhealthy organs or tissue regeneration and formation of new blood vessels. Thus, in the present review we aim to describe these approaches, advances, obstacles and opportunities as well as their application in regeneration of heart as a prominent angiogenesis-dependent organ.

## Introduction

Regenerative medicine is a branch of medical sciences with the aim of damaged restoration, malfunctioned, or missed tissue by the use of a broad spectrum of technologies and methodologies such as; tissue engineering techniques, cell transplantation approaches, stem cells (SCs) biology, biomechanics, prosthetics, and nanotechnology.^1,2^ Tissue engineering hires a mixture of cells and bio-degradable scaffolds for tissue formation. Lately, according to the several material-based approaches, the bio-degradable materials fundamentally functionalize with cellular activities.^3^

According to the researches hypotheses, the promotion of vascularization and angiogenesis is a crucial step in construction of tissue-engineered grafts.^4^ In therapeutic angiogenesis the new vessels are generated by restoration of blood flow to the ischemic tissues. Due to the lack of endogenous tissue perfusion, the ischemic diseases need this strategy to accelerate the vascularization in tissue-engineered grafts through the transplantation of *in vitro* -generated tissue for damaged or surgically treated tissues.^5,6^

Therapeutic angiogenesis emphases on restoration of original blood flow in ischemic tissues by angiogenesis regulating factors. However, inadequate and low-speed process of vascularization in tissue-engineered grafts is considered as an obstacle that limits the application of these factors.^7^ In fact, whereas the *in vitro* engineering clinical-sized tissue grafts are applicable by the use of autologous progenitors into appropriate biomaterial scaffolds, but *in vivo* implantation provides engraftment and differentiation alone in outer layer due to limited diffusion of oxygen and nutrients from vessel beds. Consequently, the lack of active vascular ingrowth leads to transplant rejection due to the necrosis in the depth of few millimeters in tissue-engineered grafts.^8^ For the successful development and progression of blood vessels into the transplanted tissues, the induction of pro-angiogenic signaling pathways is essential. Furthermore, as a prerequisite factor needed for the normal function of transplanted cells and/or acquisition of new phenotypes, the regulation of angiogenic switch is considered as a vital phenomenon. The angiogenesis occurs by the balance among the pro- and anti-angiogenic factors with cytokines. Thus, in situ production of pro-angiogenic factors is associated with vessels regeneration based on the tissue requirements.^8^ Therefore, the induction of regulated angiogenesis may provide the ability to create a transplanted tissue with high resemblance to host ones.

This article aims to review the angiogenesis process and the related mechanisms, different strategies used for the restoration of vascular structure in a distinct milieu and advantages as well as their limitations.

## Angiogenesis

### Angiogenesis definition

Angiogenesis is a morphogenic process in which new blood vessels are formed from pre-existing ones. It is a phenomenon with high importance in pathophysiology of wound healing, tissue repair, pregnancy, and exercise. Tumor formation as a result of uncontrolled vascular organization returns to the angiogenesis aberration that occurs by epigenetic factors, nucleotide polymorphisms, or endocrine irregularities.^9^ Angiogenesis mechanism in tumor cells is resembles to the normal angiogenesis. However, there are some differences not only in terms of architecture but also in the molecular expression level and its regulation. Tumor vessels are abnormal and in addition to the endothelial cells (ECs), tumor cells exist in their walls. Furthermore, the most of the tumor vessels are leaky due to the absent of functional pericytes for covering them.^10^ Moreover, it has been shown that there are some important factors that involved in angiogenesis induction in tumor cells but are not very effective in normal conditions including Ang-2, IL-1β, heparinize and, etc.^10^

Since, the angiogenesis is the most vital process involved in evolutionary changes and tissue homeostasis, efficient regulation can lead to progress in treatment of organs and tissues with deprived vascularization. Moreover, the successful modulation of angiogenesis can lead to decrease in mortality rate and increase in drug efficacy in diseases associated with angiogenesis like cancer.^11-15^

Angiogenesis is a complex process that normal, stable, and functional vessels will form according to the coordinated interplay in the space and time of various cell types and growth factors.^16^

### Angiogenesis induction by growth factors

Angiogenesis process is initiated by the activation of soluble growth factors such as vascular endothelial growth factor (VEGF), basic fibroblast growth factor (bFGF), platelet-derived growth factor (PDGF), transforming growth factor-β, keratinocyte growth factor (KGF), hepatocyte growth factor (HGF), Ephrin-B2, and angiopoietin. Angiogenesis induction relies on the balance between stimulatory and inhibitory factors of angiogenesis towards pro-angiogenic factors. The list of some important pro and anti- angiogenic factors with their related receptor is given in Table 1. According to the previous research, growth factor-based treatment leads to enhancement in the speed of granulation tissue formation, tissue generation, and wound closure.^17^ Among these factors, the VEGF-A is the most prominent regulator of both physiological and pathological angiogenesis which enables the activation of pathways leading to neovascularization when used individually.^18^ The importance of other members of VEGF as specific EC growth factors have been proved in therapeutic angiogenesis. VEGF165 injection led to enhanced revascularization in rabbit ischemic of hind limbs. Moreover, VEGF stimulated the formation of collateral vessels, increasing the blood flow and tissue perfusion relied on the presence of ECs. For these reasons, the VEGF has been exploited for progress in wound healing in diabetic animal models.^19^

**Table 1:** Pro- and anti- angiogenic factors and its receptors^17^

**Pro-angiogenic factors**	**Receptors**
VEGF	(VEGFR1, VEGFR2 and VEGFR3)
PDGF	(PDGFRα and β)
FGF	(FGFR1, FGFR2, FGFR3, and FGFR4)
EGF	EGFR (ErbB1, HER1), ErbB2 (HER2), ErbB3 (HER3) and ErbB4 (HER4)
TGF	Serine/threonine kinase receptors (type I and type II)
KGF	FGFR2b
HGF	c-Met
TNF-α	(TNFRI and TNFRII)
ANGPT-1,2	(Tie-1 and Tie-2)
**Anti-angiogenic factors**	**Receptors**
TSP1 and TSP2	CD36 Integrin associated protein (IAP, CD47)
Angiostatin	avb3 integrin
INF-α, β	IFNAR1, IFNAR2

ANGPT-1,2: Angiopoietin 1,2; EGF: Epidermal Growth Factor; EGFR: Epidermal Growth Factor Receptor; FGF: Fibroblast Growth Factor; FGFR: Fibroblast Growth Factor Receptor; HER: Human Epidermal Growth Factor Receptor; HGF: Hepatocyte Growth Factor; INF-α, β: Interferon α, β; IFNAR: Interferon-α/β receptor; KGF: Keratinocyte Growth Factor; PDGF: Platelet Derived Growth Factor; PDGFR: Platelet Derived Growth Factor Receptor; TGF: Transforming Growth Factor; Tie: Tyrosine kinase with immunoglobulin-like and EGF-like domains; TNF- α: Tumor Necrosis Factor- α; TSP1,2: Thrombospondin 1, 2; VEGF: Vascular Endothelial Growth Factor; VEGFR: Vascular Endothelial Growth Factor Receptor.

According to the essential role of angiogenesis, various approaches along with direct use of growth factors have been published as the following concepts.

## Stem cell therapy for vascular induction

SC-based therapies are one of the encouraging approaches in regenerative medicine and angiogenesis induction.^20,21^ In this regard, the SCs can be differentiated into target cells and mimicking the architecture of the original tissue, or secreted desired factors to desired position.^22^ So, the SCs can be used as gene or protein carriers.^22,23^ From the perspective of regenerative medicine, there are three types of applicable SCs including; embryonic SCs (ESCs), adult SC and induced pluripotent SCs (iPSCs).^24^

### Embryonic stem cells for vascular regeneration

ESCs are natural pluripotent SCs with high differentiation potential to all types of the body cells.^25^ Human-derived embryonic SC (hESC) are isolated from inner cell mass of a blastocyst in *in vitro* fertilization method and they are able to be differentiated into vascular ECs, smooth muscles, and cardiomyocytes.^26-28^

One of the important characteristics of ESCs is their potential for prolonged undifferentiated proliferation in culture and their capacity to differentiate into ECs by continuous maturation steps.^29^ ESC-derived ECs (ESC-ECs) reveal endothelial surface markers (CD31), express endothelial proteins (VWF and PECAM [platelet and EC adhesion molecules]), fetal liver kinase 1 (Flk 1), platelet EC adhesion molecule, and represent endothelial functions (consumption of acetylated low-density lipoprotein and formation of capillary tubes in Matrigel). Furthermore, the ease of ESCs genetic manipulation made them a proper tool to endogenous genes targeting, which can facilitate the gene expression manipulation in ESC-ECs. So, ESCs are a good renewable source to produce genetically manipulable microvascular ECs.^30^

Based on the animal studies, the application of ESC-ECs and mural cells in vasculature of the ischemic limb or myocardium have been proved, but there are ethical concerns following ESC transplantation regarding the high possibility of teratoma formation and the potential of immunogenicity.^23^ In spite of all the advantages, there are no applicable methods in regenerative medicine because of following reasons.^23^ Since, the embryo destruction occurs following isolation of ESCs, the separation is conducted without donor’s approval, thus there is still debate upon eligibility of this procedure.^27^ Furthermore, these cells are used in a heterogeneous body that is considered as an allogeneic process with the possibility of immunogenicity reaction. The incidence of teratoma formation is probable due to pluripotent nature of ESCs.^26^ Even though the establishment of hESCs banks demands high cost and time, but it contains immune phenotype cell lines which may obviate the rejection difficulties.^26^ However a number of clinical interventions with a goal of ESC transplantation are happening according to Clinical trials (www.clinicaltrial.gov).

### Induced pluripotent stem cells are involved in vascular growth

In 2006, development of iPSCs by Yamanaka’s research team^31^ has made a breakthrough evolution in regenerative medicine specifically in the field of cardiac and vascular regeneration. The iPSCs are embryonic stem-like cells with pluripotent properties in which in the laboratory conditions they are reprogrammed from differentiated state into undifferentiated. Regarding this advantage, it is possible to generate the pluripotent SCs from the adult somatic cells and further differentiation into any types of cells in order to use in tissue regeneration.^27^

Experimental studies have confirmed the ability of iPSCs to differentiate into main cardiovascular components, including smooth muscle cells, ECs, vascular mural cells, and cardiomyocytes. However, there are considerable challenges in the use of iPSC-ECs for clinical trials like the selection of the most efficient approach in order to induce the pluripotency property. Nevertheless, as discussed in the case of ESCs, the pluripotency feature is considered as a double-edged sword due to the risk of teratoma formation.^25^

Since, the angiogenesis and formation of ECs are essential steps in the field of regenerative medicine, both differentiation from iPSCs or trans-differentiation based on somatic cells into ECs could be helpful. In this case, the Cochrane and colleague conducted the differentiation of mouse and human iPSCs to the ECs^32^ and also the Sayed and colleague applied trans-differentiation of fibroblast into the EC using a small molecule.^33^

Generally, in spite of beneficial findings of iPSCs manipulation, they are not still prevalent in regenerative medicine due to the absence of a high-efficient protocol for iPSCs production and the presence of poor delivery strategies of iPSCs and iPS-derived cells to the target tissues.^34^ Vascular induction requires more precise and efficient protocols along with proper cell markers in order to reproducibility of differentiate/trance differentiate iPS/settle cells into ECs. Because dysfunction of iPS-derived ECs could potentially be contributed to the vascular inflammation and vascular thrombosis. Also, tumor angiogenesis, pathological retinopathy, and progression of atheromatous plaque could be mentioned as “off-target” effects derived from iPS-derived ECs.^24^

### Adult stem cells involved in vascular growth

Adult SCs exist in many parts of body e.g. bone marrow (BM), skeletal muscles, brain, pancreas, liver, skin, and adipose tissue. There are two different states of adult SCs including active and dormant with a high variety in SC niches called microenvironment.^23,35^ Presence of the active SCs in the body lead to repair, regeneration, and maintenance of tissue.^35^ In fact, every creature should be able to regenerate itself and the life of a creature is dependent on the regeneration process, but its degree is varied.^27^ In comparison with lower vertebrates, the adult human body has limited regenerative potential.^36,37^ Thus, a main purpose of regenerative medicine is to switch the adult SCs from dormant to an active state^35,38^ via both biochemical (i.e. growth factors) and biophysical (electrical and mechanical) stimulators.^22^ Despite the economic cost of this approach than the cell-based therapy,^27^ the cellular transplantation is dominant due to its potential to migration, secretion of paracrine factors, cell-cell interaction and the tissue regeneration.^22^ In this regard due to the autologous nature of adult SC transplantation, all advantage of this method is related to the lack of transplantation rejection.^25^ These cells are readily accessible and could be isolated and harvested from the BM, peripheral blood,^24^ or tissues such as adipose,^39^ brain, skin, and muscle.^25^ However, some disadvantages has limited adult SC clinical use.^25^ Since the *ex vivo* isolation and expansion of autologous cells is a time-consuming process, thus the delays occur in the treatment procedure.^24^ Also, these cells are not abundantly found in adult tissue and there is no successful isolation method, thus their isolation is a labor-intensive process. Another disadvantage of adult SCs administration refers to reduced number of cells that normally occurs under certain conditions like disease and aging.^25^

In 1997, Asahara discovered a subpopulation of vasculogenic endothelial progenitor cells (EPCs) derived from adult SCs which were culminated in vascular regeneration.^40^ According to a conventional definition, there are two subtypes of EPCs including hematopoietic and non-hematopoietic. The hematopoietic EPCs are subpopulations of BM-derived mononuclear cells and are derived from hematopoietic SCs, myeloid precursors and mesenchymal SCs. They are generally named as CD34+ /CD133+ /VEGF receptor 2 (VEGFR2)+ cells.^41^ The non-hematopoietic EPCs are derived from various types of tissues (i.e. adipose tissue, spleen, placenta, and blood vessel wall). They are determined with high variability in the expression of surface antigens. Angiogenic potential of BM derived-EPCs has been proved as a strategy for cell therapy.^42^ Thus, according to the published studies, the *left ventricular* remodeling and improvement of regional perfusion occurred following transplantation of CD34+ /CD133+ cells in elderly anterior myocardial infarction (MI) patients.^24^

Ischemic symptoms have been improved in various trials expressing the role of EPCs in treatment of critical limb ischemia. However, the results of meta-analyses have been questioned the promising long-term clinical results obtained from EPC-based approaches. Preclinical studies and clinical trials have provided information about the possibility of EPC therapy as a secure method to cure the ischemic cardiac and cerebrovascular diseases, while evidence indicating benefits on hard clinical outcomes are uncertain. Despite the outcomes support the EPCs in diabetic retinopathy, there are no/few results gained from randomized clinical trials to confirm it. Whether the EPCs therapy may limit the premature atherosclerosis and reduce the cardiovascular risk in inflammatory rheumatic diseases need to further investigation.^43,44^

An interesting idea for induction of angiogenesis by circulating EPCs in the target tissue is designed based on the “EPC-capture stent” as a fishing hook for trapping EPCs^24^ or even peripheral blood-derived mesenchymal stem cells (MSCs).

In BM, there are MSCs as non-hematopoietic cells beside the hematopoietic cells.^25^ According to the International Society for Cellular Therapy, there are three criteria in utilization of MSCs as follow (1) adherence to plastic during cell culture (2), an ability to expose a group of surface antigens, and (3) an ability to differentiate into various types of multipotent SCs.^45^

BM-MSCs constitute a small fraction of the total nucleated cells in the stromal compartment of BM. Moreover, they reside in other tissues like adipose tissue, skeletal muscle,^25,26^ dental pulp, and gingiva.^23^ MSCs are also present in peripheral blood (PB-MSCs or circulating MSCs) in a lower population.^45^ The tendency of MSC to home in unhealthy tissues^21,45,46^ helps the damaged tissue to be repaired by production of high amounts of bioactive trophic factors resulting in induction of parenchymal cells.^45,46^ They also present immunosuppressive and anti-inflammatory features that make them appropriate for clinical cell therapy approaches.^45^ In spite of favorable MSC-based therapy techniques, increased survival rate and reduced loss of transplanted cells, and preservation of adequate vascular supply are problems needed to be solved. Furthermore, high safety of MSC-based treatment is an advantage of this approach^45^ although there is still an incidence of tumor or ectopic tissue formation.^47^ Because of their capacity to differentiate into endothelial, pericytes and smooth muscle, these cells represent less lineage restriction and more plasticity than EPCs.^24^ Thus, their application in angiogenesis induction could be promising. Sometimes the quality of autologous MSCs is not appropriate due to the presence of comorbidities or advanced age,^45^ thus the preconditioning of SCs could be useful. For example in an animal study, the diabetic adipose–derived SCs were preconditioned with deferoxamine to restore the neovascularization potential through HIF1α pathway.^39^

While the application of SCs is faced with various issues, high amount of evidences support that transplanted SCs can locally secrete growth factors, cytokines, and chemokines when the related region is harmed as a protection impact.^48^ For example, a new strategy has been emerged based on paracrine action of MSCs for tissue repair, in which the exosomes as the potential therapeutic paracrine factors play a key role in various kinds of disease models such as cutaneous wound healing and MI.^48^ Thus, it was proved that the exosomes as paracrine regulators in SCs pathways operate based on cell-free system with no challenges that are found in whole cell transplantation method.

## Exosomes and vascular growth induction

Exosomes are extracellular vesicles of endosomal origin, secreted by any type of cell, ether normal or pathologic, and mirroring cellular content. They have small size, 30–100 nm.^23^ They are different from apoptotic bodies and other extracellular vesicles^47^ and their cargo does not constitute random molecules from the cell but precisely and specifically chosen for the delivery of the desired message and their function is mainly as mediators of intercellular communication.^23^

**Figure 1:**
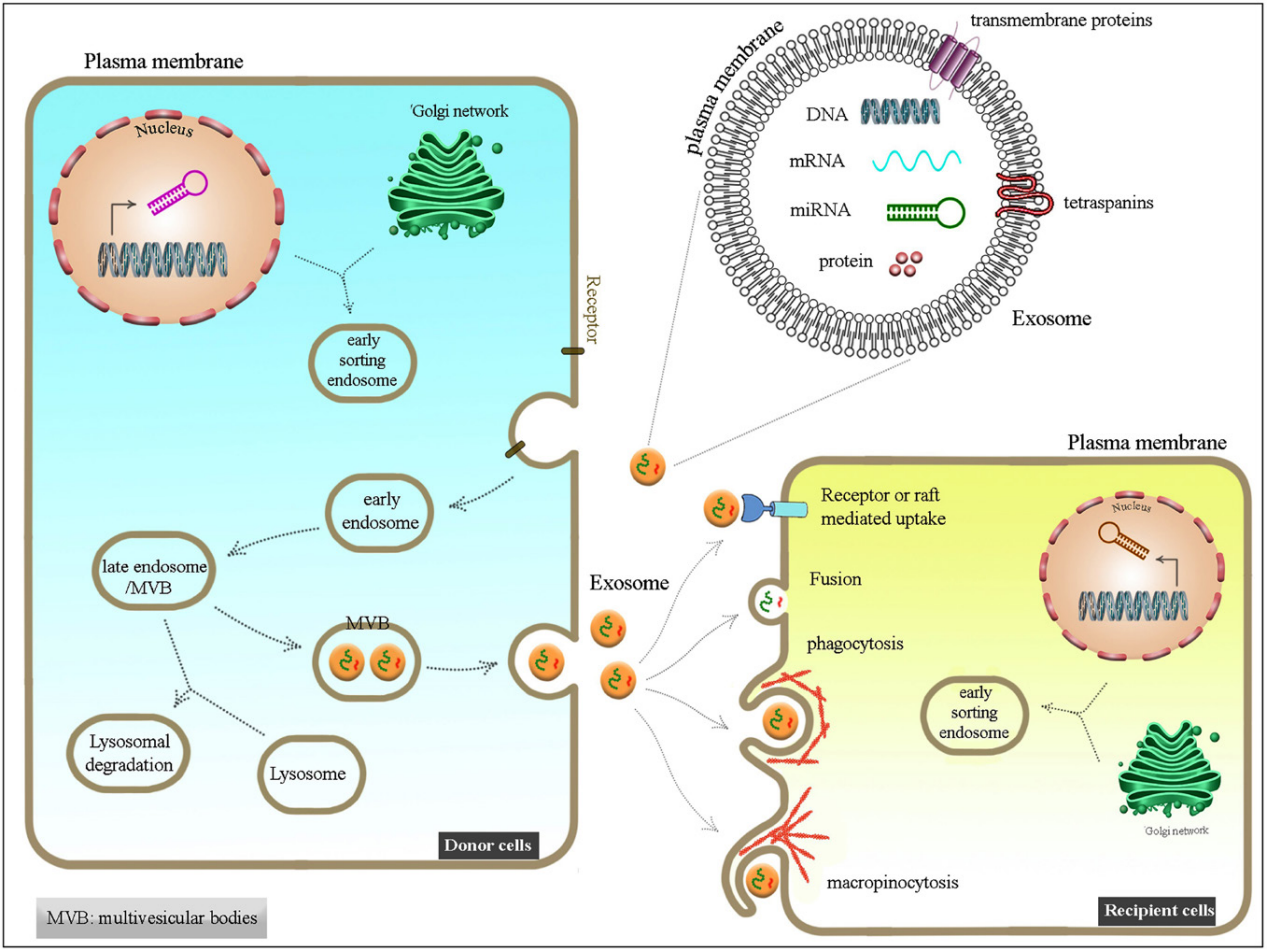
Overview of exosome formation mechanism by donor cell and its effect on the recipient one.

Biochemically they contain lipids, proteins, nucleic acids, mostly miRNA, mRNA, and sometimes genomic and mitochondrial DNA.^47^ They carry certain substances that are originated from a signaling pathway^23^ (Figure 1).

The immunomodulatory and regenerative features of exosomes are great pivotal factors. The use of SC-derived exosomes in order to formation of new blood vessels and tissue repair is conducted by generation of a balance between proliferation induction and apoptosis inhibition in recipient cells including epithelial cells and fibroblasts.^23^ In this regard, the exosomes are cell-free therapeutic candidates for cardiac regeneration and wound healing. The angiogenic effects of ESC-derived exosomes (ESC-Exos) and the exosomes derived from human EPCs have been applied MI model and wound healing process in mice, respectively.^49^

Despite all the benefits, some major challenges are mentioned. Since the cell type plays a key role in determining the contents of exosomes, the proper SCs should be employed in therapeutic approaches.^23^ Furthermore, some clinic-related issues such as finding a standard exosomes isolation method in large scale prevent the success in this method.

## Scaffolds affect the vascular growth

Challenges of basic approaches like growth factor delivery, cell transplantation, and exosomes have led to creation of new combinatorial strategies with some improvements like scaffolds. Scaffolds are biological materials (biomaterials) for the promotion of cell growth and tissue regeneration. Scaffolds form a temporary structure leading to tissue growth and formation of 3D biological environment. This biological environment in a small scale could enable the cells to grow, differentiate and gain specific phenotype. Scaffolds are classified into two types of natural and synthetic forms.^50,51^

The substrates of the natural extracellular matrix are provided by explicit diameter and highly dynamic particles which have complex interplay with cells. Scaffolds are made by natural materials to get a potential restoration rate including ligands specified for cellular connection, cell migration, and various growth factors.^51^

Collagen, fibrinogen, tropoelastin, hyaluronic acid and glycosaminoglycans are natural polymers could be found in extracellular matrix. These polymers can provide biocompatible and bioactive scaffolds and are involved in cell-cell attachment as well as arranging a niche for regulating the cell function.^18^ Other natural polymers derived from plant, insect, animal-like cellulose, chitosan, silk and fibroin have potential to produce biomaterial and provide suitable sites for cell attachment and other properties available in microenvironments for SC delivery. Not only the restrictions such as laborious sterilization and purification, high variability, and high incidence of contamination with pathogens in used polymers, but also physio-chemical properties and degradation rates of these materials are difficult to be controlled as well.^52,53^

The materials such as hydrogel of poly N-isopropyl acrylamide, poly (amidoamine), poly (vinyl alcohol), poly-L-lactic acid, and poly lactic-co-glycolic acid (PLGA) are examples of biodegradable synthetic polymers. They have some advantages like controllable chemical and mechanical properties as well as degradation rate that enables them as a promising substitution as natural materials. Thus, a variety of applicable synthetic polymers have been produced for almost all types of tissue engineering approaches.^54-56^

Scaffolds should have three important feature including biodegradability as well as enough stability to function as three-dimensional (3D) spacers. Furthermore, they should be biocompatible and can support angiogenesis. The host tissue response to the scaffold is not only dependent on its 3D structure but also the material composition may affect the success of the engraftment.^57^ So that, because of inflammation induction initially after the implant of scaffold which can be a threat factor for successful engraftment of tissue-engineering constructs, creation and select the suitable scaffolds must be important goal to control the host-tissue response after scaffold implantation.^58^

The specific materials that are used for scaffold production are important factors in determining the immune response. For example a low inflammatory response has been shown after use of alginate as a not ECM derived hydrogels. However, ECM derived materials such as collagen can have a pro-inflammatory effect. In the case of synthetic scaffolds their hydrophobic materials type such as poly (lactide-co-glycolide) (PLG) can also have inflammatory effect because of cell attachment to them via adsorbed proteins.^59^

So, nowadays these inherent inflammatory properties of scaffolds are used to design of materials for immunotherapy, as well as inflammation-driven vascularization. PLG as one of the scaffold that is used for immunotherapy (cancer immunotherapy) purpose can enhance the maturation and cytokine production of murine dendritic cells.^60^ Furthermore, it has been shown that PLG scaffold can induce vascularization and greater vessel formation than hydrogels such as collagen-chitosan-hydroxyapatite likely due to their different inflammatory effect. So that, inflammation induction by PLG is mildly than that of hydrogels which the less controlled response to their inflammation effect can result in the apoptosis of surrounding immune cells.^57^ In addition to the base material of scaffolds the cross-linking chemistry can also have an effect on immune responses. For example, it has been demonstrated that collagen gels that cross linked by glutaraldehyde can induce a greater amount of vascularization than unmodified form because its effect on macrophage infiltration is greater than unmodified collagen gels.^61^

Thus type of the used material for organs restoration and angiogenesis is depends on tissue compatibility and stiffness rate. As a result, tissue consistency and strictness of defect are used to select the proper material.

### Scaffold and growth factor delivery

Due to the high clearance and/or degradation of the factors from the environment, the administration of a specific growth factor has been always carried out with a high dosage. Extracellular matrix is physiologically responsible for modulation, control, local concentration, partitioning, bioavailability, and signaling of angiogenic growth factors.^18^ The function of biomaterial matrices along with certain types of growth factor-binding sites originated from ECM have made it possible to recapitulate their regulatory functions. The combination of growth factors with scaffolds could accelerate the rate of regeneration by presentation of growth factors to the related target sites.^62^ In order to improve the local delivery of growth factors in therapeutic angiogenesis approaches, different sorts of biomaterials with various physical and chemical features have been developed. Employment of hydrogels scaffold in ischemic tissue is a good example due to the high resemblances to the ECM, convenient process under mild conditions and proportionally low invasive delivery method.^63^ Moreover, the property of hydrogels to be degraded in accordance with the angiogenesis process has made it a unique biomaterial for pro-angiogenic matrices.^63^ Both natural (e.g. collagen, gelatin, and fibrin and polysaccharides such as hyaluronic, alginate, and chitosan) and synthetic (like polyethylene glycol and PLGA) hydrogel matrices have been produced with a great variety of delivering angiogenic factors. Due to inherent kinetics and different features for the control of growth factor release, more fundamental experiments are needed to address the underlying mechanisms. Of note, the structure and function of proteins must be preserved during the procedure.^18^

### Scaffold and cell delivery

Cell delivery in the process of tissue regeneration and angiogenesis induction occurs more than 90% in 24h clearance from the transplantation sites with a maximum value of 1% in 4-week post-transplantation.^64^ Due to the high risk of the processes, non-invasive methods are utilized. In several years of investigations, scholars have attempted to find an efficient SCs delivery method with the highest therapeutic effects. What is considered as the most prominent goal is the right homing of transplanted cells into the ischemic areas and increase the survival and retention of cells following transplantation. Furthermore, increasing the angiogenic potential in host tissue will make it possible to modify the cell behavior post-transplantation.^65^ In this case, the scaffolds are reliable components providing various intra-structural growth factors, cell attachment basement, and developing a 3D platform for cell-to-cell communication. These characteristics have made the scaffolds an accurate candidate for efficient angiogenesis and cell adaptation after transplantation.^65^

It is confirmed that alteration of physicochemical and morphological properties of cells in various types of scaffolds could be a potential modulator in angiogenesis.^67^ The reciprocal crosstalk among the cells and adjacent scaffolds mediated by adhesive molecules like laminin, fibronectin, vitronectin, tenascin, and hydrophilic proteoglycans triggers the explicit kind of signaling called external transduction. On the other hand, the attachment of cell to surfaces occurs by expression of integrins, immunoglobulin superfamily, cadherins, selectins, and other adhesives molecules.^67^ Juxtacrine interaction of cell receptors with cognate motifs in scaffolds, various intracellular biochemical reactions would be ignited, resulting in the modulation of cells phenotype, motility and migration, dynamic cell growth and genes expression. Thus, not only the cell-matrix interaction is maintained, but also the cell-cell communication is improved.^68^

Scaffolds that are used for delivery of cells or growth factors in the field of regenerative medicine are mainly different transplantable (demands host surgery) and injectable (less invasive) factors. The injectable factor is the most important advantage in development of angiogenesis.^69^

Natural ECM originated from decellularized organ is an ultimate carrier for cell transplantation and helps vasculature system and complex architecture retain almost like to the original organ.^4^ Lately, it has been shown that the ECM could serve both as substrate for cellular attachment and migration and also a binding domain for growth factors, including VEGF, fibroblast growth factor (FGF), and HGF.^70^ According to these features of decellularized ECM, it is an appropriate biophysical and physiological milieu for loaded cells. Up to date, different types of organs have been decellularized successfully, including heart, liver, urinary bladder, skin, lung, tendon, and blood vessels. Moreover, the success has been reported in trials with simulation of animal disease models such as urinary bladder, skin, heart, and lung.^71^ Taylor et al discovered that the recellularization process by cardiac cells or ECs into the cardiac ECM are derived from decellularization via the perfusion. The biomimetic tissues could preserve the functional contraction and electrically stimulated in vitro for 28 days.^72^

Functionalized synthetic polymers, such as polylactide, polyglycolide, and their copolymer (PLGA), as well as hydroxyapatite can be produced via serum proteins (e.g. fibronectin or vitronectin), thus they could provide binding sites for cell adhesion. Cells with/without growth factors could be captured in such scaffolds with significant scales and need surgery for transplantation.^73,74^ Similarly, scaffold-based delivery of transfected cells with up-regulated VEGF in various animal models enable a promotion in angiogenesis, bone formation, and vasculature.^75^ MSCs that inherently overexpress the VEGF are proper sources for cell transplantation in wound healing.^76,77^

## Angiogenesis in cardiac tissue regeneration

New vascular development is necessary for regeneration of some organs with high dependence to the angiogenesis including cardiac tissue engineering.^78^ Thus, in the next section, the engineering and regeneration process of the heart reviewed by referring to the approaches mentioned in this article.

### Cardiac tissue regeneration

Cardiovascular disorders especially MI and peripheral artery disease are related to high morbidity and mortality rates worldwide. Unsuccessful l*eft ventricular* remodeling and heart failure followed by MI are contributed to a lowered life expectancy of the patients, thus the urge for restoration of ischemic injury and muscle recovery is essential.^79^

The application of cardiac tissue in regeneration of large muscle lesions in MI or other myocardial disorders is not plausible due to the restricted regeneration feature in cardiac tissues. Following the first clinical sign of injury, a thick collagenous scar is formed by fibroblasts and ECs function. Despite the protective characteristic available in structure of cell, the issues like its inflexibility and non-contractile potential in constructed tissue can result in heart failure.^7^ In spite of a decrease in the extent of the myocardial necrosis in MI patients led by blood flow recovery in the infarcted region (through the initial restoration of the obstructed epicardial coronary artery), there is still compliances among some other patients.^80^ Thus, as a substitution, the inhibition of dysfunction in microvascular system of ischemic heart is used in order to myocardial restoration. In this regard, the angiogenesis is considered as an approach to apply reperfusion in ischemic myocardium immediately following MI and also prolonged remodeling of left ventricular that inhibits heart failure.^79^

Cardiac transplantation is the most prevalent surgical approach for restoration of the cardiac tissue. But the presence of some challenges has limited its extensive application such as the lack of availability of donor organ, the need for prolonged immunosuppression intervention and utilization of anticoagulants.^81^ Currently in order to angiogenesis simulation and restoration of myocardial function, the regenerative medicine and tissue engineering have been introduced some alternatives agents.^82^ In this case, growth factor delivery, cell therapy (injected cells to animals or patients) and the use of various biomaterials have been introduced as novel MI treatment approaches.

### Cells and growth factors effect in cardiac regeneration

In order to induce heart regeneration, various kinds of cells have been discovered like BM mononuclear cells, ESCs, MSCs, skeletal myoblasts, EPCs, and cardiac SCs. Among those, the EPCs and MSCs are appropriate candidates to be used for angiogenesis induction via secretion of growth factor or the direct differentiation into the cells responsible for angiogenesis.^83,84^

The MSCs in cardiovascular repair could be injected either systemic or locally. Studies have shown that systemically injected MSCs migrate to the infarcted myocardium at various time points which could be differentiated into a cardiac phenotype cells or participate in angiogenesis in the infarcted area.^1,85^ Also SC delivery to the infarcted border zone in MI patients showed greatly improved tissue perfusion and enhanced total left ventricular function. These cells could be used either in autologous or allergenic manner, because of their immunomodulatory properties.^45^ As mentioned previously, the VEGF is the most prominent growth factor used for angiogenesis induction and cardiac regeneration with the ability of injection to the ischemic area. However, due to leaky and unstable properties of blood vessels in using of this factor alone, some others such as FGF and PDGF are used together.^86^

There are some limitations for effective SC therapy in cardiac regeneration which the major problems are the cells type selection for therapy and their effective delivery as well as limited maintenance of the cells in the heart, and the risk of immune rejection. So that, significant cell death and cell removal from the heart after direct injection of SCs into the heart muscle has been shown. However it has been demonstrated by preclinical studies that intravenous MSCs therapy after acute MI can improve the left ventricular function and decrease the adverse remodeling in mice with large infarcts.^87^

### Exosomes in cardiac regeneration

Exosomes play a vital role in rectification of the cardiac function by improving the angiogenesis, and inhibition of the fibrotic tissue formation and apoptosis. In a study, treatment of mouse ESC-derived exosomes (ESC-Exos) with miR-294, enhanced the endogenous repair and preserved the heart function in a MI mouse model. Moreover, it is believed that the preservation of the heart function is related to the enhanced angiogenesis, decreased apoptosis and increased cardiomyocyte proliferation by partial mediation of miR-294.^88^

It is determined that the regeneration of cardiosphere-derived cells (CDCs) occurs mainly by mediation of exosomes that are rooted from. Furthermore, the CDC-derived exosomes including selective administration of considerable amounts of microRNA-146a simulates the effective functions of CDC exosomes.^49^ Currently, the security and efficacy of allogeneic CDCs delivery in MD patients via a multi-vessel intracoronary delivery in patients with cardiomyopathy secondary to Duchenne MD are in progress as a phase I/II clinical trial named HOPE-Duchenne (Halt cardiomy- OPathy progrEssion in Duchenne; NCT02485938). While the cell-derived exosomes have less immunogenicity than the cell transplantation, but inherent immune responses triggered by exosomes is observed in MI patients that urges needs for comprehensive methods.^89^

Furthermore, it was found that exosomes derived from cardiac progenitor cells can induce angiogenesis and reduced fibrosis. Moreover, it has been shown cardiac progenitor cells exosomes treatment improved cardiac function, decrease cardiac apoptosis and enhanced intracardiac angiogenesis.^90^

The study of cardioprotective effect of MSC^GATA‐4^ exosomes showed that they could give anti-apoptotic miRNAs (miR‐19a) into cultured cardiomyocyte in vitro and into the ischemic myocardium in vivo. Furthermore, it has been indicated that the levels of some proteins including IGF‐1α and pAkt increased but, active caspase 3 level in cardiomyocytes was downregulated.^90^

### Scaffolds in cardiac regeneration

Despite the prosperous function of cell therapy and growth factor injection in cardiac function in both animal and human studies, the existing obstacles such as low cell retention/engraftment, poor cell delivery, electromechanical integration, and prolonged safety need to be overcome. It seems that bioengineering is a potential way to obviate these problems.^91^ As aforementioned, delivery of biomaterials to cells or growth factors can occur alone, with a scaffold or by a carrier. Clearly, administration of injectable biomaterials lead to expansion of the ventricular wall, improvement in angiogenesis and repairing of endogenous tissue as well mediated by recruited endogenous SC that finally leads to the maintenance of cardiac function. Both natural and synthetic injectable biomaterials with potential therapeutic applications in animal MI models are presented in Table 2.^79^

**Table 2:** Overview of various injectable scaffolds uses in MI^80^

**Scaffolds (Natural and synthetic)**	**Material form**	**Delivery factor**
Alginate	Hydrogel	HGF, IGF-1; PDGF-BB, VEGF-A
Chitosan	Hydrogel	bFGF; FGF-2
Collagen	Hydrogel	N/A
Decellularized myocardial ECM	Hydrogel	N/A
Decellularized pericardial ECM	Hydrogel	bFGF; HGF
Dextran	Microparticles	HGF
Fibrin	Hydrogel	bFGF
Gelatin	Microspheres	bFGF; IGF-1, VEGF
Hyaluronic acid	Hydrogel	rTIMP-3
Keratin	Hydrogel	N/A
Matrigel	Hydrogel	N/A
PEG based	Hydrogel	VEGF; HGF, VEGF; HGF, IGF-1; EPO
PLGA based	Microparticles, nanoparticles	NRG-1, FGF-1; VEGF; IGF-1
PNIPAAm based	Hydrogel	bFGF
UPy	Hydrogel	HGF, IGF-1

bFGF: basic fibroblast growth factor; FGF: fibroblast growth factor; HGF: hepatocyte growth factor; IGF: insulin-like growth factor; MI: myocardial infarction; PDGF-BB: platelet-derived growth factor BB; PEG: polyethylene glycol; PLGA: poly(lactic-co-glycolic acid); PNIPAAm: poly(N-isopropylacrylamide); rTIMP: recombinant tissue inhibitor of matrix metalloproteinase-3; SDF-1: stromal cell-derived factor; UPy: ureidopyrimidinone; VEGF: vascular endothelial growth factor.

## Conclusion and future perspectives

The generation of functional vascular networks is an important step for regenerative medicine strategies. This phenomenon is necessary for survival of engineered tissue grafts and blood flow restoration in ischemic tissues. There are several approaches for vascular regenerative therapy, and many studies have been applied in this regard including cell-based therapy and using various growth factors. However, because of some limitations in the application of the above-mentioned methods, some other ways are invented such as different biomaterials for improving the angiogenic effects and its induction in the best way. Nevertheless, using different types of scaffolds have advantages and limitations such as different stability and degradation properties as well as time for release of growth factor. Thus, uninterrupted development of the approaches may lead to discovery of new strategies for the formation of vascular network in the field of regeneration medicine especially in the cardiac system, wound healing and skin regeneration. Therefore, because of some obstacles related to these strategies, multicenter, large-scale and randomized controlled clinical trials may be fundamental and mandatory. These trials by administration of best therapeutic methods should be conducted to prove the safety and efficacy of angiogenesis that finally leads to standard treatment strategy for the patients suffering from severe diseases.

## Ethical Issues

Not applicable.

## Conflict of Interest

There is no conflict of interest.

## Acknowledgments

We would like to specially thank to Prof. Ali Mostafaie, and Dr. Mohsen Rastegari-Pouyani for their valuable discussions and help with manuscript preparation. This review was supported by Medical Biology Research Center, Kermanshah University of medical sciences.
